# Bilateral Electric Cataracts With Markedly Disparate Onset Times

**DOI:** 10.7759/cureus.103030

**Published:** 2026-02-05

**Authors:** Loubna Moulahid, Hatim Bazhar, Nabil Bouslous, M.Omar Moustaine

**Affiliations:** 1 Ophthalmology, Mohammed VI University Hospital, Agadir, MAR

**Keywords:** electric cataract, electrocution, management challenges, therapeutic intervention, visual prognosis

## Abstract

Ocular injuries secondary to high-voltage electrocution are uncommon and may lead to delayed manifestations, particularly cataract formation. In this article, we describe the case of a 21-year-old male diagnosed with a unilateral electric cataract. The patient had previously undergone cataract surgery in the left eye following a high-voltage electric shock sustained at the age of seven and presented years later with decreased vision in the right eye. Clinical examination revealed an anterior subcapsular cataract in the right eye, with no other associated lesions, particularly involving the retina or the optic nerve head. Prompt therapeutic intervention with phacoemulsification surgery successfully restored normal visual acuity in the affected eye. This case underscores the critical importance of ongoing vigilance in detecting and managing ocular complications following electrocution, even years after the initial incident.

## Introduction

Electrocution is a serious occurrence that may inflict damage of varying severity to a multitude of tissues in the organism, and in some cases, may be fatal [1-3]. Electrical injuries can result in a wide spectrum of ocular complications. The reported incidence of cataract following electrical injury varies in the literature, ranging from 0.7% to 8.0% [4]. This limited number of cases may be due to the reduced survival rate of patients who have experienced high-voltage electrocution, as cataract formation requires long-term survival. In this article, we present the case of a young patient diagnosed with an electric cataract that manifested as reduced visual acuity several years after the electrocution event.

## Case presentation

Patient information and timeline 

**Figure 1:**
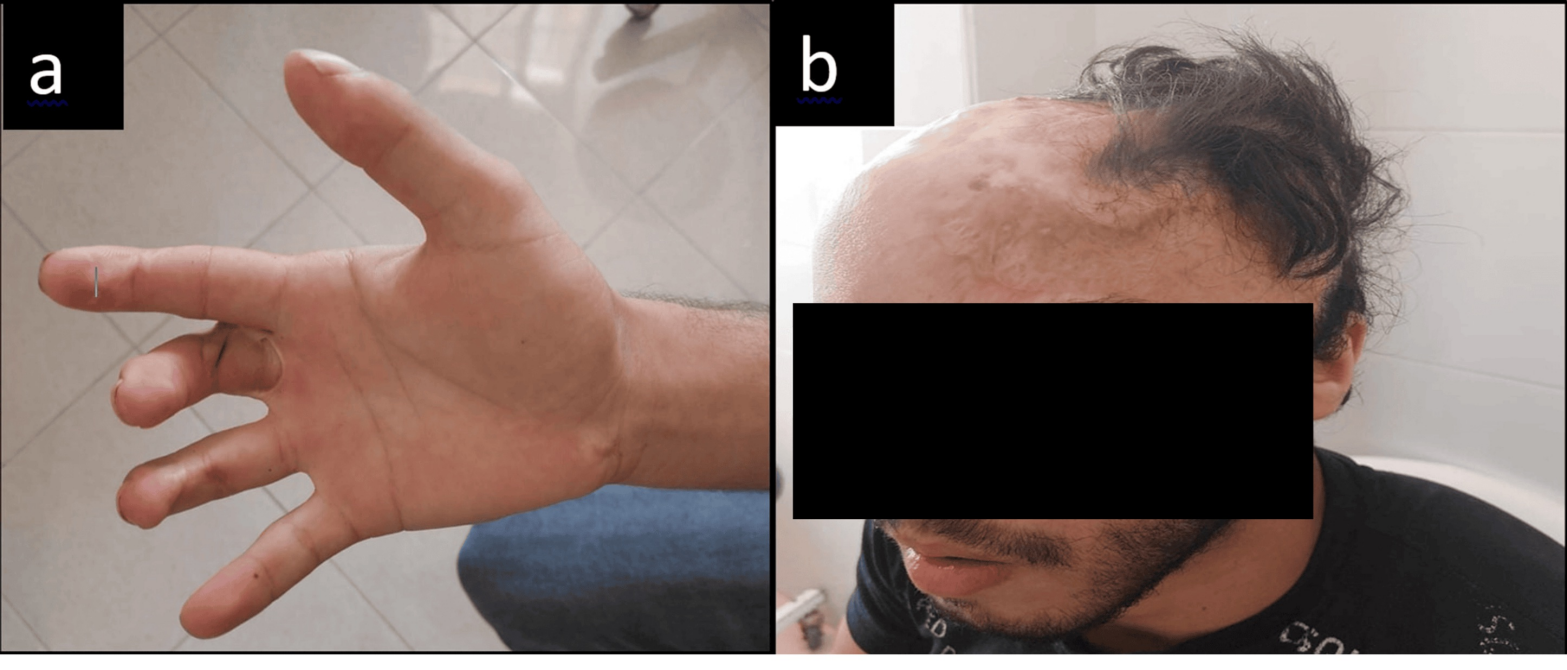
Burn scars resulting from electrical shock in the patient. The entry point (right hand) (a) and the exit point of the electric current (head) (b)

A 21-year-old African male presented to our ophthalmology department with a complaint of decreased vision in the right eye persisting for several years. At the age of seven, the patient sustained an accidental high-voltage electric shock while climbing a utility pole. Subsequently, the patient was hospitalized in the intensive care unit for approximately one month, during which burn injuries were noted on the right hand (entry site of the electric current) and approximately two-thirds of the right scalp (exit site) (Figure 1). Additionally, the patient reported a history of cataract surgery in the left eye, performed elsewhere, six years prior.

Clinical findings 

**Figure 2:**
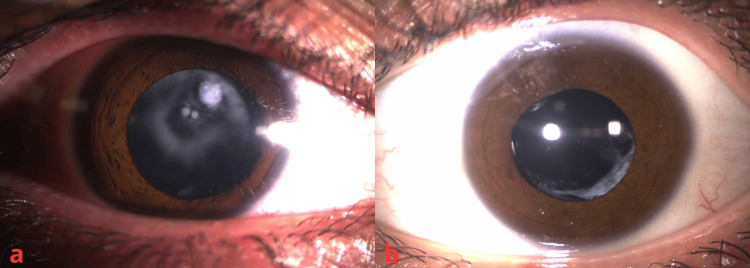
The right eye shows an anterior subcapsular cataract (a), and the left eye is pseudophakic (b)

Upon ocular examination, the best-corrected visual acuity was assessed as 20/200 in the right eye and 20/20 in the left eye. Pupillary examination revealed round, regular pupils that exhibited normal reactivity to light bilaterally. Slit-lamp examination revealed the presence of an anterior subcapsular cataract in the right eye (Figure 2), while the left eye was pseudophakic (Figure 2). Fundoscopic evaluation revealed normal findings in both eyes. Intraocular pressure measurements were within the normal range bilaterally.

Therapeutic intervention 

Under topical anesthesia using oxybuprocaine, the patient underwent an uneventful right-eye phacoemulsification surgery with the implantation of a foldable intraocular lens into the capsular bag.

Follow-up and outcome

**Figure 3:**
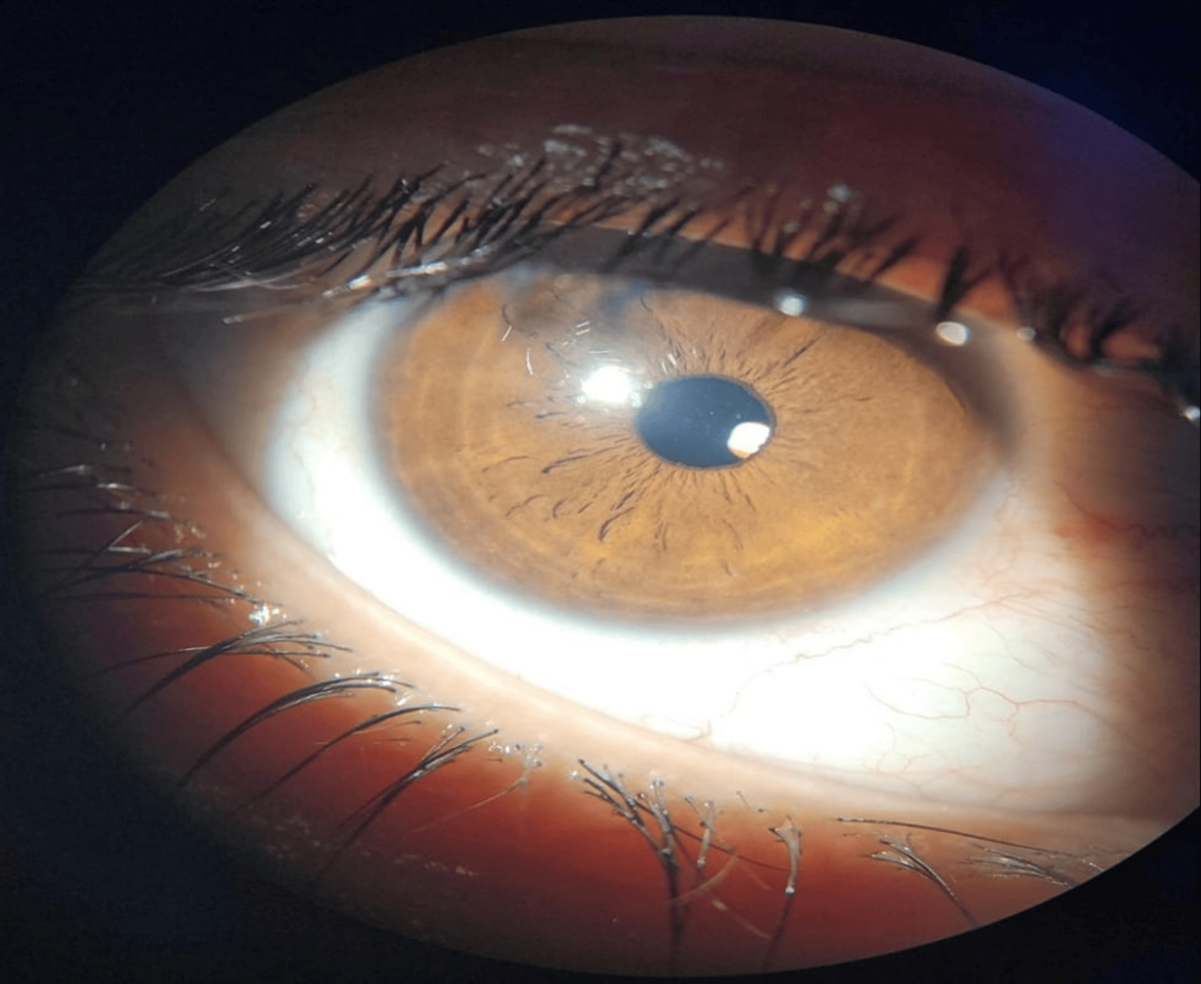
Postoperative outcome of the right eye at one month

The postoperative course was uneventful, and the patient regained a visual acuity of 20/20 (Figure 3).

## Discussion

In some low- and middle-income countries, accidental electrocution represents an important public safety concern, particularly in environments where electrical infrastructure may be insufficiently protected. One example includes the presence of overhead high-tension power lines located close to residential buildings.

In terms of ocular injuries, a range of manifestations can occur following high-voltage electrocution. These manifestations vary from mild injuries such as conjunctival hyperemia, chemosis, corneal opacities, uveitis, miosis, and cataract, to more severe ones, including retinal edema, papilledema, choroidal rupture, chorioretinal necrosis or atrophy, retinal detachment, and optic atrophy [4,5].

The exact pathogenesis of the process resulting in cataract formation remains unclear. Various hypotheses have been proposed; some suggest a direct electrocoagulative effect on the proteins composing the lens material, while others believe that mechanical damage to the lens caused by powerful ciliary contraction at the moment of electric shock is the causative mechanism. Additionally, hypotheses implicating decreased permeability of the lens capsule and nutritional disturbances of the lens due to iritis cannot be disregarded [2,6]. Other causes that may result in a similar morphology, such as blunt ocular trauma, inflammatory cataract, or metabolic disorders, were considered less likely in this case due to the absence of relevant clinical history.

The development of cataract may occur either immediately after the electric insult or following a latency period that can extend for several years post-trauma [1,5]. In early-onset cases, this diagnosis was considered only after the exclusion of other potential causes of cataract formation.

Regarding the progression of cataract, it may exhibit a prolonged stationary phase or slowly advance, eventually leading to the formation of total or subtotal cataracts [1,4,5,7].

The varying onset times of cataract development in both eyes following an electric shock can be explained by differences in progression rates. Logically, the eye closer to the electric entry point (the right eye) would experience more immediate and pronounced damage, but cataract formation in that eye may progress more slowly. In contrast, the more distal eye may have a delayed onset of cataract formation but progress more rapidly, potentially causing significant visual disturbances and leading to earlier surgical intervention [8].

In the present case, the cataract was predominantly anterior subcapsular, a morphology that has been classically described in electrically induced cataracts. Previous studies have reported that the earliest lens changes following electrical injury consist of multiple fine vacuoles located beneath the anterior subcapsular region, typically in the mid-periphery of the lens. Over time, these vacuoles may evolve into flake-like opacities that gradually coalesce and migrate toward the visual axis, leading to clinically significant visual impairment [4,5].

As in our case, the anatomic and functional outcomes of electrically induced cataract managed by cataract extraction and posterior chamber intraocular lens implantation are highly favorable, provided that there are no concomitant ocular lesions such as optic atrophy, chorioretinal lesions, or retinal detachment [8-10]. However, the lack of additional imaging or functional testing may limit the ability to detect subtle optic nerve or chorioretinal abnormalities. In addition, photographic documentation of the left-eye cataract prior to surgery was not available, which represents a limitation of this report.

## Conclusions

Electrical injury is an uncommon etiology of cataract. Upon stabilization of the patient’s condition, it is crucial to conduct a meticulous ophthalmological evaluation for every survivor of an electrocution accident to detect potential ocular injuries. A normal initial examination should not negate the importance of long-term follow-up, as electric cataracts may develop months or years after the incident in one or both eyes.
